# Cancer incidence following a high-normal platelet count: cohort study using electronic healthcare records from English primary care

**DOI:** 10.3399/bjgp20X710957

**Published:** 2020-07-28

**Authors:** Luke TA Mounce, Willie Hamilton, Sarah ER Bailey

**Affiliations:** University of Exeter Medical School, Exeter.; University of Exeter Medical School, Exeter.; University of Exeter Medical School, Exeter.

**Keywords:** blood platelets, cancer incidence, epidemiology, primary care

## Abstract

**Background:**

A raised platelet count (thrombocytosis) measuring >400 × 10^9^/l is associated with high cancer incidence. It is uncertain whether platelet counts at the upper end of the normal range (high-normal: 326–400 × 10^9^/l) are also associated with cancer.

**Aim:**

To investigate cancer incidence following a normal platelet count in primary care.

**Design and setting:**

A prospective cohort study was undertaken using data from the Clinical Practice Research Datalink and National Cancer Registration and Analysis Service, dating from 1 May 2005 to 30 April 2014.

**Method:**

One-year cancer incidence was estimated for 295 312 patients with normal platelet counts (150–400 × 10^9^/l). Patients with platelet counts >325 × 10^9^/l were oversampled to maximise precision of estimates of cancer incidence. All patients were aged ≥40 years with no prior cancer diagnoses. The effects of age, sex, and smoking were explored. Non-melanoma skin cancers were omitted from exclusions and incidence.

**Results:**

One-year cancer incidence increased greatly with age, male sex, and higher platelet count. Males aged ≥60 years with a high-normal count had an incidence of 4.2% (95% confidence interval [CI] = 4.0 to 4.4). The highest incidence of 6.7% (95% CI = 5.3 to 8.4) was found in males aged ≥80 years, who had platelets in the range of 376–400 × 10^9^/l; this was 3.1 percentage points higher than the incidence for patients in the same age group with lower-normal counts of 150–325 × 10^9^/l. Risks for all female subgroups were <3%. Patients with high-normal platelet counts were most at risk of lung and colorectal cancers and, in general, had advanced-stage cancer at diagnosis.

**Conclusion:**

Platelet counts at the high-normal range in males aged ≥60 years may be indicative of an underlying malignancy, and referral for further investigation should be considered.

## INTRODUCTION

Raised platelet count (thrombocytosis) is a newly discovered marker of cancer in primary care; the 1-year incidence of cancer in patients with thrombocytosis has been found to be 11.6% for males and 6.2% for females.^1^ These figures far exceed the 3% threshold set by the National Institute for Health and Care Excellence (NICE) for investigating possible cancer in the UK.^2^ Even marginally raised platelet counts are associated with a clinically relevant increased risk of cancer,^3^ although the underpinning mechanism(s) for the platelet–cancer association are yet to be fully characterised.

Platelet count varies with age and sex,^1^^,^^3^^–^^5^ ethnicity,^6^^,^^7^ and has a genetic component.^8^ Despite proposals for tailored reference ranges for different ages and sexes,^5^ current UK guidance on interpreting platelet count applies a uniform threshold of 400 × 10^9^/l to all patients.^2^^,^^9^

Identifying the platelet count at which patients are at a ≥3% risk of cancer can contribute to the improved selection of patients for further investigation in primary care and, crucially, avoid unnecessary investigation in those with lower risk. As such, the authors examined cancer incidence, overall and by site, in patients with a normal platelet count, stratified by age and sex, with particular focus on those with counts at the upper end of the normal range.

## METHOD

### Data sources

Electronic primary care medical records for this prospective cohort study were extracted from the Clinical Practice Research Datalink (CPRD) GOLD database. CPRD contains anonymised electronic records, including all patients’ consultations, diagnoses, and laboratory results from approximately 8% of UK practices. Linkage to data held by the National Cancer Registration and Analysis Service (NCRAS) was available for all patients from English practices; this provided a second mechanism for identifying cancer diagnoses and the stage of cancer at diagnosis.

### Patient samples

Eligible patients had a platelet count of 150–400 × 10^9^/l recorded between 1 May 2005 and 30 April 2013; were aged ≥40 years at the time of the platelet count; and registered at a practice with NCRAS cancer registry linkage. Two samples were extracted:

- patients who had a platelet count at the upper end of the normal range (high-normal), classified as 326–400 × 10^9^/l. Their index date was defined as the date of their first high-normal platelet count, even if this was not their earliest recorded count. This emphasis on sampling patients with a high-normal platelet count was undertaken to maximise power for subgroup analyses. These patients were further grouped by platelet count: 326–350 × 10^9^/l (high–normal 1); 351–375 × 10^9^/l (high–normal 2); and 376–400 × 10^9^/l (high–normal 3); and
- a comparison group of patients with no recorded high-normal platelet count (lower-normal). Their index date was that of their first count in the range of 150–325 × 10^9^/l.

**Table:** How this fits in

The risk of cancer in primary care patients with thrombocytosis (an elevated platelet count of >400 × 10^9^/l) has been found in males and females to be almost four and two times above the 3% threshold for urgent investigation for suspected cancer set by the National Institute for Health and Care Excellence, respectively. The authors investigated patients with a platelet count at the upper end of the normal range (high-normal: 326–400 × 10^9^/l) to help determine whether cancer should be considered. It was found that older males with a high-normal platelet count have an increased incidence of cancer within 1 year compared with those with a count that is well within the normal range. At the upper end of the normal range, colorectal cancer was most likely to be diagnosed in males and so, in the absence of any other indicative clinical features, a faecal immunochemical test may be the most appropriate initial investigation. These findings support the usefulness of platelet count as a clue to identifying patients who could be harbouring a cancer.

Patients were excluded if they had a cancer diagnosis prior to the index date or a subsequent cancer detected through screening.

### Patient characteristics

Sex and age at index date (categorised in 10-year bands up to ≥80 years) were retrieved from CPRD records. Smoking status (‘never’ or ‘past/current’) was identified using Read code and product code (for smoking cessation therapies) lists published by Booth *et al.*^10^ Patients’ records were examined for alarm symptoms of cancer in the 21 days prior to their index date.^3^

### Outcome variables

Incident cancer diagnoses in the year following the index date were identified by searching patients’ CPRD GOLD records for any of the 2182 cancer-related Read codes covering 21 specific sites in the body, plus a miscellaneous category. This list of Read codes (available from the authors on request) has been utilised in many previous studies. Incident diagnoses for the same period were also extracted from NCRAS data using 02 (morphology) codes from the 10th revision of the International Classification of Diseases. As is standard practice, non-melanoma skin cancers were not studied; as these are largely diagnosed visually, identifying blood-based markers is not likely to be clinically useful. Diagnoses recorded in either the CPRD or NCRAS data were accepted, with the earliest record in either assigned as the diagnosis date. Stage at diagnosis was extracted from NCRAS data, where available, and was dichotomised as ‘early’ (stages 0–2) or ‘advanced’ (stages 3–4).

### Sample size

To estimate a cancer incidence of 3% (NICE’s recommended threshold for urgent referral) with a margin of error of ≤1%, a sample size calculation indicated that 1118 patients were needed per age/sex/platelet count subgroup. Feasibility counts suggested this size would be reached in all strata except in males aged ≥80 years in the high-normal 3 group. Due to the size of the dataset being limited to 300 000 patients as a result of budgetary and CPRD restrictions, all eligible patients in the high-normal groups were included; the remaining allocation was given to patients in the lower-normal group, for whom an equal-sized simple random sample (SRS) was taken for each of the 10 age/sex subgroups.

### Statistical analysis

The 1-year cancer incidence is reported as a percentage (with 95% confidence intervals [CIs]), stratified by platelet count group, age band, and sex, as well as comparative baseline cancer incidence (as determined from NCRAS-reported national incidence figures). For patients in the high-normal groups, this is equivalent to the positive predictive value (PPV) for cancer of their platelet count. Incidence is presented by platelet count group for subgroups in which a significant increase in incidence was observed.

The most commonly diagnosed cancers were identified, together with the proportions of patients for whom a high-normal count was the first recorded feature of these cancers. Odds ratios (ORs) by platelet count group for diagnosis with these cancers, and any cancer, were obtained from logistic regressions that adjusted for age, sex, and smoking history; these utilised the full sample.

A further logistic regression model was constructed to investigate the association between a high-normal count and advanced stage at diagnosis, which controlled for patients’ age, sex, smoking history, and site of diagnosed cancer. This model included all patients with diagnosed cancer and complete staging information.

Analyses were conducted using Stata/SE (version 15.0), and results reported in accordance with the Strengthening the Reporting of Observational Studies in Epidemiology (STROBE) statement.^11^

## RESULTS

**Figure 1.**
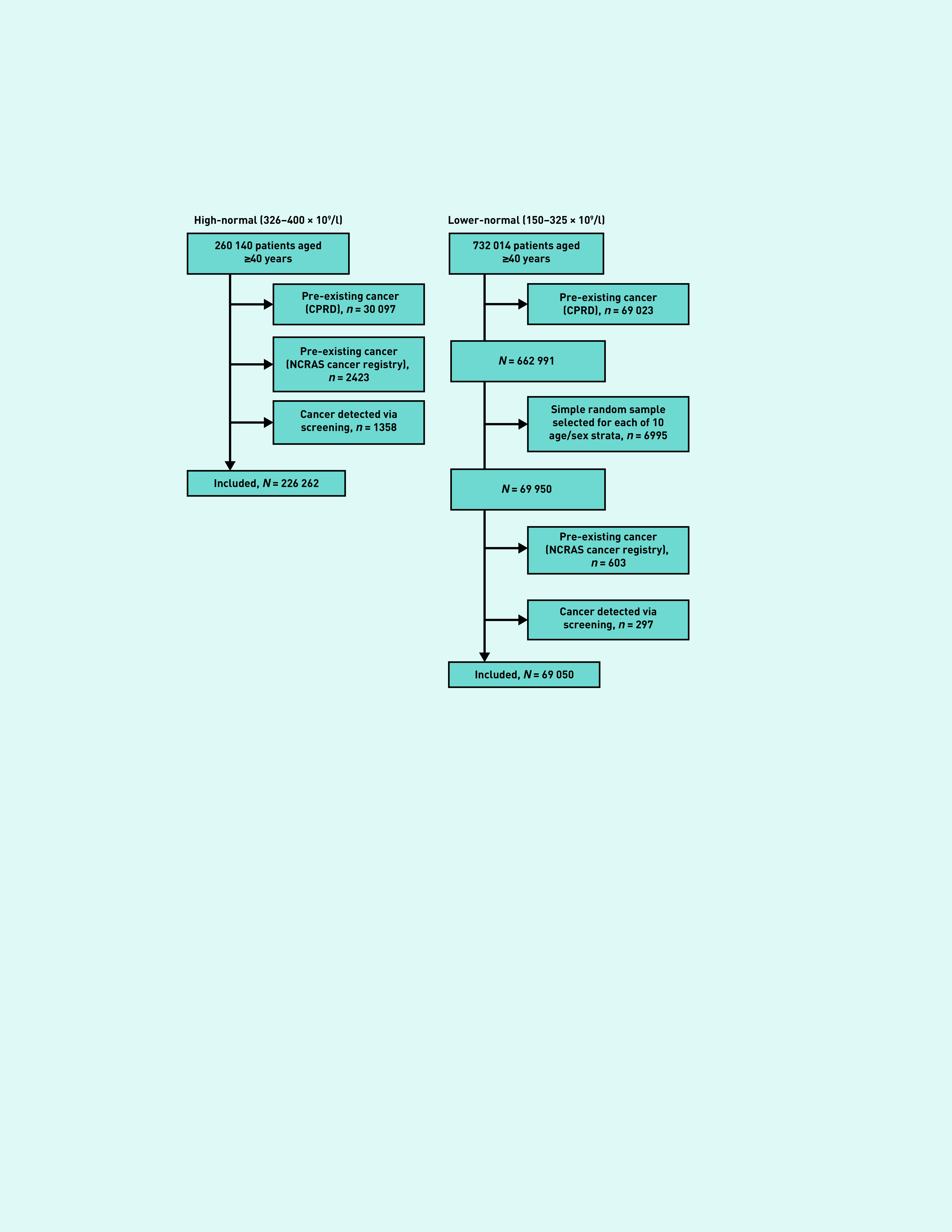
***Cohort identification flowchart. CPRD = Clinical Practice Research Datalink. NCRAS = National Cancer Registration and Analysis Service.***

The final sample consisted of 226 262 patients with high-normal counts, and 69 050 patients with a lower-normal count (Figure 1). The median age for patients in the high-normal groups was 60 years (interquartile range 47–71 years) and 158 081 (69.9%) were female. An SRS of 6995 patients in the lower-normal group was taken for each age/sex subgroup; demographics are not reported as the methods created an artificial population.

### Cancer diagnoses

A total of 5178 incident cancers were recorded in the data sources. In total, 762 were only recorded in the CPRD, 866 were only recorded by NCRAS, and 3550 incident cancers were reported in both. In 68 181 male patients from the high-normal groups, there were 1869 incident cancers diagnosed within 1 year (2.7%; 95% CI = 2.6 to 2.9). In contrast, of 158 081 female patients in the high-normal groups, there were 2206 cancers (1.4%; 95% CI = 1.3 to 1.5). The comparable incidences for patients in the low-normal group were 2.1% (95% CI = 2.0 to 2.3) for males and 1.1% (95% CI = 1.0 to 1.1) for females. Regression models showed increases in the risk of cancer with increasing platelet count, adjusted for sex, age, and smoking status (Table 1).

**Table 1.** Regression models predicting 1-year incidence of all cancers, lung cancer, and colorectal cancer^a^

**Covariate**	**All cancers, OR^b^ (95% CI)**	***P*-value**	**Lung cancer, OR^b^ (95% CI)**	***P*-value**	**Colorectal cancer, OR^b^ (95% CI)**	***P*-value**
**Age, years**						
40–49 (reference)	1.00	—	1.00	—	1.00	—
50–59	1.72 (1.54 to 1.93)	<0.001	3.94 (1.97 to 7.89)	<0.001	2.61 (1.88 to 3.61)	<0.001
60–69	2.88 (2.60 to 3.20)	<0.001	9.11 (4.74 to 17.49)	<0.001	5.36 (3.96 to 7.26)	<0.001
70–79	4.30 (3.88 to 4.76)	<0.001	10.5 (5.44 to 20.26)	<0.001	9.74 (7.26 to 13.08)	<0.001
≥80	4.30 (3.86 to 4.79)	<0.001	7.55 (3.74 to 15.23)	<0.001	9.44 (6.94 to 12.84)	<0.001

**Sex**						
Female (reference)	1.00	—	1.00	—	1.00	—
Male	2.01 (1.90 to 2.13)	<0.001	1.63 (1.26 to 2.09)	<0.001	2.01 (1.77 to 2.29)	<0.001

**Smoking status**						
Non-smoker (reference)	1.00	—	1.00	—	1.00	—
Past/current smoker	1.01 (0.94 to 1.07)	0.868	3.71 (2.41 to 5.70)	<0.001	0.88 (0.77 to 1.01)	0.079

**Platelet count (× 10^9^/l)**						
150–325 (reference)	1.00	—	1.00	—	1.00	—
326–350	1.36 (1.26 to 1.47)	<0.001	3.84 (2.38 to 6.19)	<0.001	2.60 (2.10 to 3.22)	<0.001
351–375	1.56 (1.44 to 1.70)	<0.001	4.49 (2.73 to 7.37)	<0.001	3.76 (3.02 to 4.68)	<0.001
376–400	1.63 (1.49 to 1.79)	<0.001	4.68 (2.79 to 7.87)	<0.001	3.93 (3.12 to 4.97)	<0.001

a Each model used data from all 295 312 patients.

b ORs of >1 indicate increased odds of cancer relative to the reference group; ORs of <1 indicate decreased odds. CI = confidence interval. OR = odds ratio.

**Figure 2.**
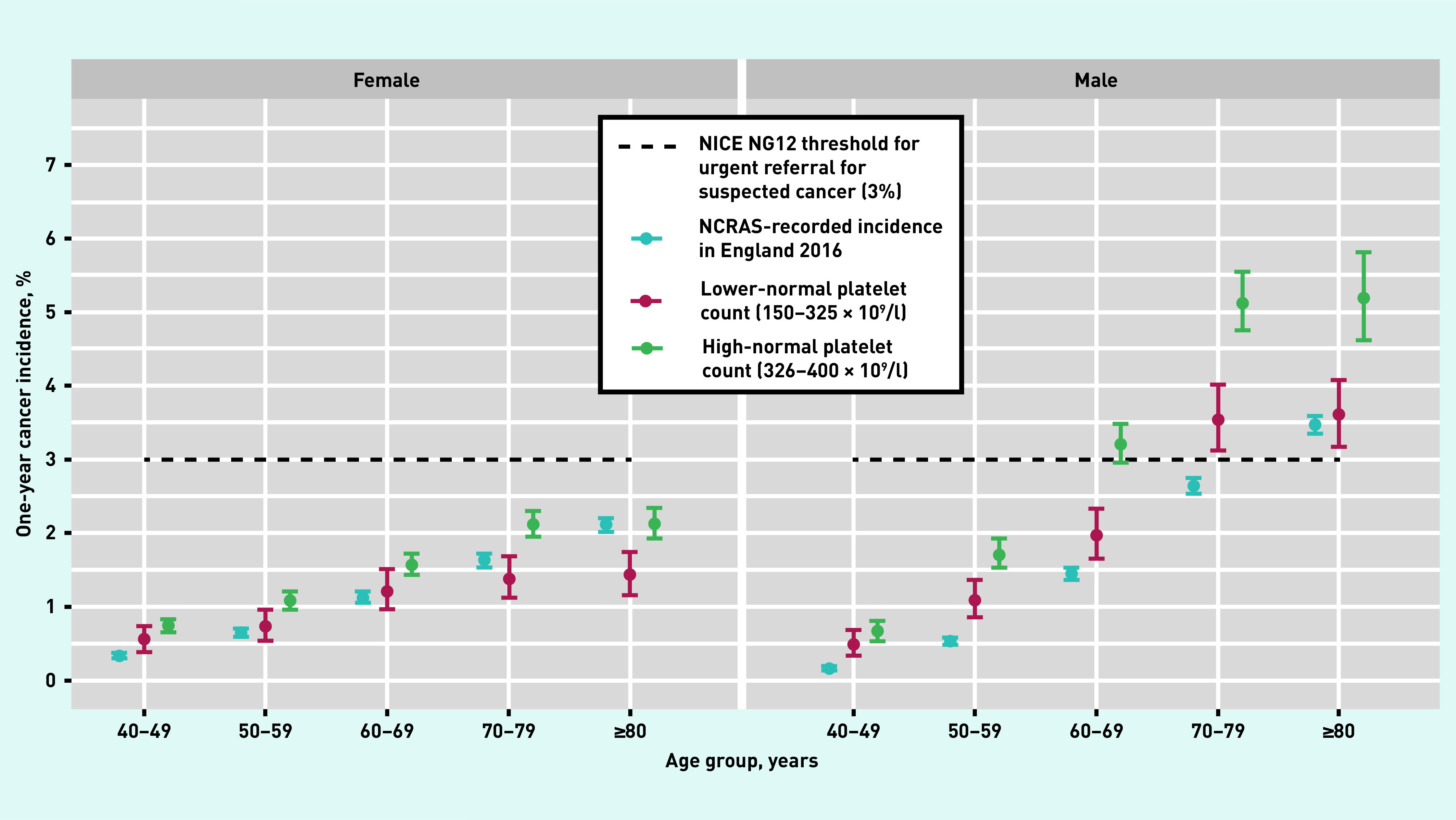
***One-year incidence of all cancers (excluding non-melanoma skin cancers) across age and platelet groups. NCRAS = National Cancer Registration and Analysis Service. NICE = National Institute for Health and Care Excellence.***

Figure 2 displays 1-year incidence of cancer by age band for patients in the high-normal and lower-normal groups, together with NCRAS-recorded incidence rates for England in 2016 (when NCRAS was formed in a merger of the National Cancer Intelligence Network and National Disease Registration), stratified by sex. Incidence increased with age for both males and females, although no female subgroup’s incidence surpassed the 3% NICE risk threshold; for that reason, females were excluded from subsequent descriptive analyses. For males, however, significantly higher incidences were evident for patients with a high-normal count relative to low-normal counts for all but the youngest age group.

Table 2 displays the 1-year cancer incidences for males stratified by age band and platelet count (corresponding incidences for females can be found in Supplementary Table S1). All high-normal subgroups for males aged ≥60 years met the NICE threshold for referral; among these, the lowest incidence was in 60–69-year-olds in the high-normal 1 group at 3.0% (95% CI = 2.7 to 3.4) and was highest in ≥80-year-olds in the high-normal 3 group at 6.7% (95% CI = 5.3 to 8.4); this represents an absolute increase in cancer risk of 3.1 percentage points over males with lower-normal platelet counts in the same age group. Overall, the incidence for a high-normal count for males aged ≥60 years was 4.2% (95% CI = 4.0 to 4.4).

**Table 2.** One-year cancer incidence (95% CI) for males by age band and platelet group, with comparable national incidence

**Age band, years**	**National incidence**	**Platelet group**			

		**Lower-normal: 150–325 ×10 ^9^/l**	**High-normal 1: 326–350 ×10 ^9^/l**	**High-normal 2: 351–375 ×10 ^9^/l**	**High-normal 3: 376–400 x 10^9^/l**
40–49		*n*= 6984^a^	*n*= 8230	*n*= 4492	*n*= 2617
	0.2 (0.1 to 0.2)	0.5 (0.3 to 0.7)	0.7 (0.5 to 0.9)	0.7 (0.5 to 1.0)	0.5 (0.3 to 0.9)
50–59		*n*= 6976	*n*= 9523	*n*= 5223	*n*= 3070
	0.5 (0.5 to 0.6)	1.1 (0.9 to 1.4)	1.7 (1.4 to 2.0)	1.6 (1.4 to 2.0)	1.9 (1.4 to 2.4)
60–69		*n*= 6953	*n*= 9286	*n*= 5122	*n*= 3138
	1.5 (1.4 to 1.5)	2.0 (1.7 to 2.3)	3.0 (2.7 to 3.4)	3.2 (2.8 to 3.7)	3.8 (3.1 to 4.5)
70–79		*n*= 6915	*n*= 6206	*n*= 3620	*n*= 2186
	2.6 (2.5 to 2.7)	3.5 (3.1 to 4.0)	4.7 (4.2 to 5.3)	5.8 (5.1 to 6.6)	5.3 (4.4 to 6.3)
≥80		*n*= 6851	*n*= 2826	*n*= 1582	*n*= 1060
	3.5 (3.4 to 3.6)	3.6 (3.2 to 4.1)	4.7 (4.0 to 5.6)	5.1 (4.0 to 6.3)	6.7 (5.3 to 8.4)

a n *is the size of the respective male age/platelet stratum.*

### Site of diagnosed cancers

**Figure 3.**
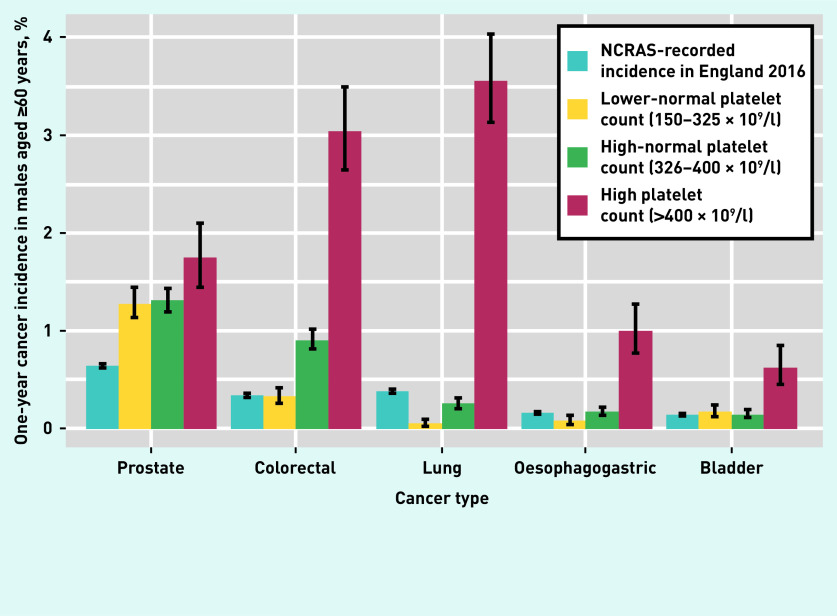
***One-year incidence for the most common cancer sites in males aged ≥60 years. Vertical solid black lines indicate 95% confidence intervals. Data for ‘High platelet count> (>400 × 10^9^/l)’ group taken from the dataset described in Bailey* et al*.****^1^* ***NCRAS = National Cancer Registration and Analysis Service.***

For males aged ≥60 years in the high-normal groups the most common incident cancers within 1 year were prostate, colorectal, and lung (Figure 3). Two less-common cancers, oesophagogastric and bladder, are also shown; these appear to only have an association with platelet counts of >400 × 10^9^/l. Including all 295 312 patients, logistic regressions (Table 1) showed that a platelet count of 376–400 × 10^9^/l was associated with odds nearly five times greater than that for patients in the lower-normal group for lung cancer (OR 4.68; 95% CI = 2.79 to 7.87), and nearly four times greater for colorectal cancer (OR 3.93; 95% CI = 3.12 to 4.97).

Sensitivity analyses for the all-cancer model confirmed that:

- the magnitude of effect of having a high-normal platelet count did not differ between males and females (interaction OR 0.98, 95% CI = 0.85 to 1.12) (data not shown); and
- effects did not substantially alter when outcomes were restricted to NCRAS-recorded cancers only.

### Other features of cancer

Of males in the high-normal groups aged ≥60 years, 65 (74.7%) of the 87 diagnosed with lung cancer, and 164 (68.9%) of the 238 diagnosed with colorectal cancer, had no recorded alarm features of cancer in the 21 days before their index platelet count.

### Stage at diagnosis

Of 4416 NCRAS-recorded incident cancers, 1855 (42.0%) had staging information: 720 (38.8%) of those indicated that the cancer was advanced. Missingness was random with respect to platelet count. After controlling for age, sex, and smoking status, a high-normal count represented an increase of nearly 50% in the odds of advanced stage at diagnosis (OR 1.5; 95% CI = 1.22 to 1.97) (data not shown).

## DISCUSSION

### Summary

This study of nearly 300 000 patients with a normal platelet count (150–400 × 10^9^/l) in primary care found the 1-year incidence of any type of cancer increased in male patients aged ≥60 years who presented with a platelet count of ≥326 × 10^9^/l. For female patients, the risk of cancer did not meet the UK’s agreed threshold for urgent investigation in any age/platelet-count group, although cancer became more common in both sexes with higher platelet counts. Patients with high-normal counts were at 50% increased risk of advanced-stage cancer at diagnosis.

The odds of being diagnosed with cancer were up to 4.7 times higher for lung cancer and 3.9 times higher for colorectal cancer for those patients who had high-normal counts relative to the comparison group.

### Strengths and limitations

Key strengths of this study are the size of the cohort, its primary care setting, and its prospective design, allowing for precise and reliable estimates of cancer incidence in the clinical setting where suspicion of cancer most commonly arises. The platelet results were transferred electronically to patient records in the CPRD, minimising missing data and possible recording errors. The NCRAS linkage is also a strength: 82.3% of CPRD-recorded cancers were also recorded in NCRAS. Only 768 of the total 5178 (14.8%) cancers were recorded solely in the CPRD, a figure similar to that found in other studies.^1^

Blood tests in UK primary care are generally ordered when a clinician wants to explore possible reasons for a patient’s reported ill health. This selects a population somewhat more ‘ill’ than the untested population. The increased cancer incidence in the sample presented here relative to the expected value (from national incidence figures) will, in part, reflect this. However, the study design included a comparison sample with the same blood test taken, allowing the authors to identify absolute risk increases; this may explain the different histograms in Figure 3.

Prostate cancer incidence was higher in those who were tested than in the NCRAS recorded data for the general population, essentially irrespective of the platelet result. This suggests little or no true association between high platelet counts (or thrombocytosis) and prostate cancer. In contrast, a lower-normal platelet count has the same colorectal cancer incidence as the general population, but the incidence is higher with high-normal counts, and much higher with thrombocytosis. Even more notable is the lung cancer–platelet relationship, in which low-normal platelet counts provide some genuine reassurance; the cancer risk is lower than baseline with such a result. However, high-normal and thrombocytosis results suggest a considerably increased lung cancer risk.

One limitation is that the PPVs reported here are for high-normal counts following primary care testing, but the indication for the blood test was unknown — GPs rarely record reasons for blood tests. A second limitation is that it was not possible to study ethnicity as this was poorly recorded in the CPRD.

### Comparison with existing literature

To the authors’ knowledge, this is the first study to report cancer incidence stratified by age and sex for patients with high-normal platelet counts. Cancer incidences for patients with platelet counts within the normal range were lower than those reported for patients with thrombocytosis.^1^ Age,^4^ ethnicity,^6^^,^^7^ and other genetic factors^8^ influence the platelet count; a number of studies have called for updated age-, sex-, and ethnicity-based platelet reference ranges,^4^^–^^6^^,^^12^ but no primary care-based studies have taken these factors into account when examining platelet counts and cancer diagnosis to date. The study presented here supports the argument that it would be clinically useful to adopt different reference ranges for platelet count, based on age and sex.

In the present study, lung and colorectal cancers were particularly associated with high-normal platelet counts. The lungs are a site of especial importance in platelet production, perhaps contributing to the findings.^13^^–^^15^ Colorectal cancer is frequently accompanied by bleeding into the gastrointestinal tract, perhaps provoking increased platelet production, although the authors were unable to study this.

### Implications for research and practice

This study found that females with a high-normal platelet count are at low risk of possible cancer, and the incidence of cancer in these groups falls below the thresholds stipulated by NICE guidance^2^ that warrant further investigation. However, that does not mean that platelet count cannot be diagnostically useful in females. Females have higher platelet counts than males, primarily due to the effects of oestrogen on platelet-forming processes in the bone marrow;^16^ the higher baseline platelet count in females means that any increase in accompanying malignancy will likely occur over the currently accepted ‘normal’ threshold of 400 × 10^9^/l.

There is no evidence for differential cancer–platelet effects in the two sexes. Observed changes in platelet count, rather than the absolute count, may be a more clinically useful measure for the early detection of cancer. This has not been fully explored in observational research studies and should be a focus for future research.

The PPVs inform clinicians of the cancer-related implications of a high-normal platelet count result, irrespective of the reason for testing. However, they should not be interpreted as evidence to support measuring a platelet count specifically to identify, or refute, cancer. In effect, the high-normal results in males should be seen as a marker of possible cancer in a similar way to a symptom of possible cancer.

Given the current accepted upper range of ‘normal’ platelet count of 400 × 10^9^/l for adults in the UK, and in the absence of any national initiative to change that, the key clinical implication from this study is that a platelet count of >325 × 10^9^/l in males, and any unexplained increase to >400 × 10^9^/l in females, should prompt clinicians to consider possible cancer. The higher risk ratios associated with lung and colorectal cancer mean that faecal immunochemical testing and chest X-ray are reasonable first investigations in the absence of any other symptoms that may suggest a specific cancer or alternative diagnosis.

## Funding

This research was funded by the National Institute for Health Research (NIHR) Policy Research Programme, conducted through the Policy Research Unit in Cancer Awareness, Screening and Early Diagnosis (grant reference number: 106/0001). The views expressed are those of the authors and not necessarily those of the NIHR or the Department of Health and Social Care. This research also received support from the CanTest Collaborative, which is funded by Cancer Research UK (grant reference number: C8640/A23385).
